# Optical Capacitance/Conductance-Voltage Characteristics of Stored Charges in Organic Light-Emitting Diodes

**DOI:** 10.3390/molecules25122818

**Published:** 2020-06-18

**Authors:** Chengwen Zhang, Zheng Xu, Peng Wang, Zilun Qin, S. Wageh, Ahmed Al-Ghamdi, Suling Zhao

**Affiliations:** 1 Department of Physics, Faculty of Science, King Abdulaziz University, Jeddah 21589, Saudi Arabia; zhangchengwen6@126.com (C.Z.); aghamdi90@hotmail.com (A.A.-G.); 2 Key Laboratory of Luminescence and Optical Information, Ministry of Education, Beijing Jiaotong University, Beijing 100044, China; zhengxu@bjtu.edu.cn (Z.X.); 18126221@bjtu.edu.cn (P.W.); 16118444@bjtu.edu.cn (Z.Q.); 3 Physics and Engineering Mathematics Department, Faculty of Electronic Engineering, Menoufia University, Menouf 32952, Egypt

**Keywords:** organic light-emitting diodes, interfacial charges, stored charges, optical capacitance/conductance-voltage

## Abstract

In this paper, capacitance/conductance-voltage characteristics (C/G-V) under illumination was achieved to investigate the dynamic mechanism of stored charges in OLEDs with a structure of ITO/ PEDOT:PSS/PMMA/Alq_3_/Al. For all devices, at least two peaks presented in the optical capacitance-voltage curve. Compared to curves of devices under dark, the first peak increased remarkably with a deviation to *V_bi_*, which can be explained in the form of stored charges combined with the optical conductance characteristics. It was also found that a great decrease in capacitance is followed by the collapse of the first peak with PMMA thickness increased. It can account for the presence of interfacial charges, which is proved further by the conductance curves. To the device with 10 nm PMMA, a third peak took place in optical capacitance and it was due to the storage of electrons by PMMA. Also, the first capacitance peak enhanced approximate linearly as the illumination power increased, which can verify the contribution of the stored charges. Additionally, it shows the potential for the stored charges in optical detections.

## 1. Introduction

With increasing demands for superior performance of flat-panel display (FPD), organic light-emitting diodes (OLEDs) are considered promising for self-luminous, flexible and high-efficient display technology [1,2,3,4,5,6,7]. Especially in recent years, OLEDs have achieved remarkably in efficiency [8,9,10,11]. To get access to better performance, further understanding on basic carriers’ process in operation of OLEDs is indispensable. In general, it includes the carrier injection, carrier transportation, exciton formation and radiative recombination. During the carrier transportation, some space charges can generate in bulk such as stored charges, which are probably originated from low mobility of carriers in the devices, mismatch of energy levels and defects or traps in the materials. Stored charges not only bring great loss of carriers but also go commonly against the performance of the device due to quenching, nonradiative transition, thermal energy accumulation or even degradation [12,13,14,15,16]. Thus, it is essential to deeply investigate the mechanism of these charges and to find a way to address this issue. Presently, some ways are proposed in different aspects to reduce stored charges and make charge more balanced. In terms of device architecture, it mainly depends on boosting carrier injection and improving exciton recombination with confining carries, the former including carrier injection layer [17,18], self-assembled molecular layer (SAM) [19,20] and the latter relying on carrier blocking layers and quantum well structure. For materials, most organic materials show low mobility of electrons resulting in charge unbalanced and stored charges at the interface of organic functional layers with significant energy level difference. As a result, in the respect of material, more and more bipolar organic materials are under investigation such as thermally activated delayed fluorescence (TADF) materials [21,22,23] and hybrid locally and charge transfer (HLCT) materials [24,25,26].

The stored charges in OLEDs behave noticeably under transient condition since they are sensitive to the change of electric field. Thus, some transient approaches were used to investigate these excess space charges, e.g., transient electroluminescence( EL ) measurement [12,27,28,29,30], impedance spectroscopy (IS) [31,32,33,34,35,36], and photo charge extraction by linearly increasing voltage (photo-CELIV) [37,38,39,40], etc. Transient electroluminescence measurement is often used to study the mechanism involved are carrier capture, exciton quenching and emission by driving the device through pulses. In addition, impedance spectroscopy reveals mechanism of stored charges in devices according to the Mott-Schottky characteristic due to the modulation of the Schottky barrier. As for photo-CELIV, its ability to measure charge carrier mobility and recombination simultaneously has attracted much interest in the organic semiconductor research. Capacitance-voltage measurement (C-V), as a part of IS, has been widely used to fully analyze the behaviors of charges in organic devices [41,42,43,44,45,46,47,48,49]. In OLEDs, this measurement was carried out to study the interfacial charges existing between N,N’-Bis(naphthalen-1-yl)-N,N’-bis(phenyl)benzidine( NPB )and Alq_3_ heterojunction [41,42]. By tuning the temperature or driven frequency, it provides the access to deeply investigate the characteristics of traps in bulk [43,44,45]. Some attention was paid to the negative capacitance so as to shed light on more underlying process of carriers [33,45]. Besides, the simulation of differential capacitance can also reveal its relation to Gaussian energetic disorder for single-carrier and double-carrier devices [46]. The defect density can also be determined by capacitance under illumination [47]. Among these works, some C-V characteristics just present one peak [43,44,45], but it is also intriguing to find two peaks in some experiments [34,44,47,48,49].

In this article, C/G-V under illumination was carried out to investigate the stored charges in OLED devices. Two peaks can be found in both dark and optical capacitance, while the first peak in optical capacitance was more enhanced than that under dark as well as deviation. The optical conductance revealed the presence of stored holes. Additionally, the charges captured by interface can also contribute to the great decrease during the collapse of the first peak. Furthermore, a third peak, owing to accumulated electrons, was detected in the optical capacitance of the device with 10 nm PMMA. Under different illumination power, the behaviors of the first peak can verify the presence of stored charges, which also has the potential for the optical detection and thus provides a new way to take advantage of these harmful charges.

## 2. Experimental Details

The used materials as poly (methyl methacrylate) (PMMA), tris-(8-hydroxyquinoline) aluminum (Alq3) were purchased from Xi’an p-oled Optoelectronics Technology Co., Ltd., China. ITO substrates, Poly(3,4-ethylenedioxythiophene):poly (styrene sulfonate) (PEDOT:PSS) and aluminum (Al) were purchased from Banhetec Co., Ltd., China. All meaterials were bought and used without any purification. 

**Figure 1:**
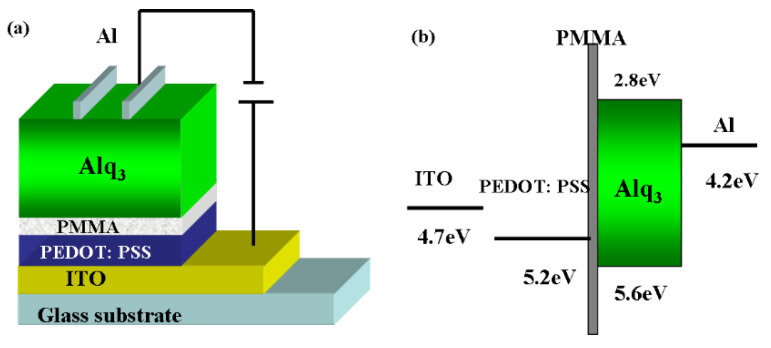
(**a**) Device structure of the OLEDs (**b**) Energy level diagram of the OLEDs.

The devices were fabricated on patterned indium tin oxide (ITO) with a sheet resistance R□ of 20 Ω. The structure was carried out as ITO/ PEDOT: PSS (20 nm)/ poly (methyl methacrylate) (PMMA) (2 nm, 6 nm, 10 nm)/ Tris-(8-hydroxyquinoline) aluminum (Alq_3_) (80 nm)/Al (80 nm), and the structure free of PMMA was the control device. The structure and the energy level diagram of the OLEDs are shown in Figure 1. The ITO substrate was cleaned with detergent water, ethanol and de-ionized water in sequence by an ultrasonic cleaner and dried with nitrogen, then followed by a UV-ozone treatment for 10 min. PEDOT: PSS was used to increase the work function of anode, which was prepared by spin-coating at 4000 round per minute (rpm) for 40 s and annealed at 150 °C for 10 min. PMMA was dissolved in chloroform and spin-coated on ITO with different speeds to form various thicknesses. Alq_3_ was thermally evaporated at pressure of 5 × 10^−4^ Pa. Then Al was sequentially deposited under a high vacuum condition of 2 × 10^−4^ Pa through a shadow mask. Their thicknesses were monitored and controlled with quartz crystal monitors. The active area of the device is 0.09 cm^2^.

The current density-voltage-luminance (*J-V-L*) characteristics were measured with a programmable Keithley Source Meter 2410 and CR-250 colorimeter. The capacitance-voltage (C-V) and conductance-voltage (G-V) characteristics were measured with the C-V unit of Keithley Source Meter 4200. The measurement frequency was 10 kHz. The rms modulation voltage amplitude was 30 mV. A solar simulator (ABET Sun 2000) with AM 1.5G was used to provide illumination with different power. All measurements were carried out at room temperature under ambient atmosphere.

## 3. Results and Discussion

**Figure 2:**
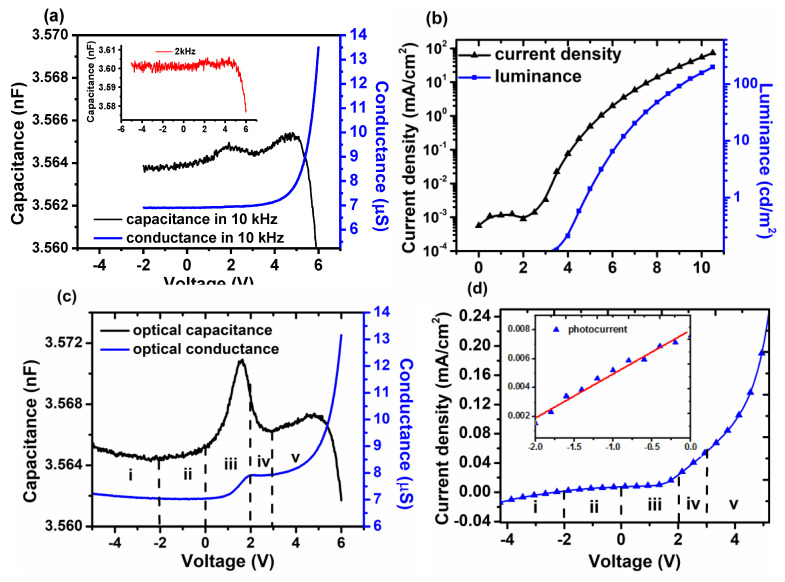
(**a**) The C-V and G-V characteristics of control device under dark, inset is the C-V characteristic in 2 kHz (**b**) The *J-V-L* characteristics of the control device. (**c**) The C-V and G-V characteristics of control device under illumination (**d**) The photocurrent of control device under illumination, inset is the photocurrent in region ii.

The C/G-V and *J-V-L* characteristics of the control device under dark are depicted in Figure 2a,b. Commonly, C-V characteristic is frequency dependent. It will weaken the capacitance in high frequency for the low response of carrier. The results of 2 kHz and 10 kHz are shown in Figure 2a. As can be seen, the capacitance in 2 kHz is too rough to resolve. Although the capacitance in 10 kHz was weakened, the peak located bias maintain unchanged. Thus, C-V for all devices operated in 10 kHz. Obviously, two peaks can be seen in capacitance with their peak pointing around 2 V and 4.7 V, respectively. It was reported that the first peak represents the depletion capacitance in correlation to the built-in potential *V_bi_^44^*. Herein, thus 2 V is defined as the *V_bi_* of the control device. However, as shown in Figure 1b, *V_bi_* of the control device, which can be derived from the difference between the work function of cathode and PEDOT:PSS, is just 1 V. It can be explained for the interfacial dipole between Alq_3_ and Al so as to rise the vacuum level by 1 eV [50,51], i.e., *V_bi_* increases to 2V. The second peak, increasing at 3 V and decreasing at 4.7 V, can attribute to the excess charges deriving from injected carriers [44], known as the diffusion capacitance. As Alq_3_ is a n-type semiconductor, electrons will mainly contribute to the second capacitance. Then holes can recombine with electrons remaining in the bulk, leading to the remarkable decreasing in capacitance. To make it evident, the *J-V-L* characteristics are illustrated in Figure 2b. As it shows, the current density of the control device did not increase at low voltage until to *V_bi_*, 2 V. Then the current density first increased slowly and enhanced notably at 3 V, which resulted from the injection of electrons and would form the second capacitance. Together with the increase current, a dim luminance took place, which indicated a weak carrier recombination. When the bias increased to 4.7 V, the obvious luminance of 1 cd/cm^2^, deriving from the enhanced recombination, took place, which would eventually result in the decrease of capacitance.

Along with the capacitance, the differential conductance G defined as:

(1) $$G=\frac{dI}{dV}$$

It is commonly measured to obtain the admittance of device [33]. As G reflects the change of current flowing through the device, it can also shed light on the carrier dynamic process. Thus, we obtained the conductance of the control device as well. As the result shows, when the control device operated at reversed and low forward voltage less than 2 V, its conductance remained as a constant, which can evidently reveal the first peak is related to the depletion capacitance rather than excess charges in bulk. As the voltage was more than 3 V, G increased gradually along with the voltage as great injection of electrons took place according to equation (1). With the voltage increasing to more than 4.7 V, G continued to increase while C decreased for increasing holes. Hence, conductance G can provide a way to deeply investigate the capacitance.

With respect to the capacitance under dark, the one under illumination can exhibit some intriguing phenomenon worth studying [37]. Thus, we measured the optical capacitance, which is shown in Figure 2c,d. In contrast to the capacitance under dark, it can be found that the first peak was enhanced remarkably with its voltage corresponding to peak located at 1.6 V, deviating from *V_bi_* by 0.4 V. It is closely related to the excess carriers excited by photons. In equilibrium, the photons first give rise to excitons in bulk. For Mott-Schottky diode, the excitons may dissociate close to the Schottky barrier. Obviously, the dissociation may take place around cathode for the large barrier for electrons’ injection. The separated holes and electrons then are driven by built-in field and drifted to anode and cathode. For n-type Alq_3_, holes’ mobility is remarkably less than electrons’. Additionally, transportation in long distance will lead holes more capable of being captured by the localized states. Thus, these stored holes generated by illumination will have great influence on capacitance as space charges. To some extent, it can be taken as illumination-induced doping. The deviation of 0.4 V indicates the existence of local field *V_sp_*. It is worth noting that under the persistent illumination, the presence of these charges should contribute identically to the capacitance in the whole range. Instead, the great change occurred only in the first peak, which can attribute to the variation of field in bulk. In detail, the dissociation of excitons and transport of photo-carriers are both in close correlation to the field induced by built-in potential, applied bias and space charges, i.e., *V_bi_*, *V_app_* and *V_sp_*.

**Figure 3:**
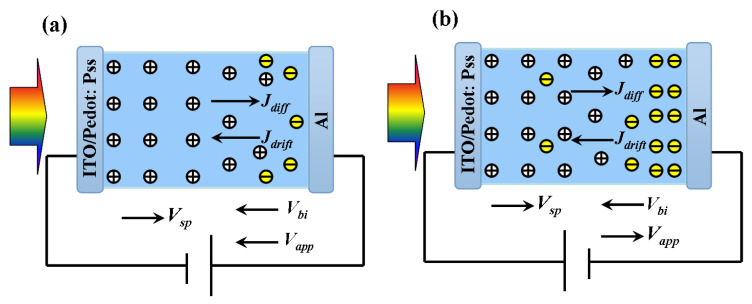
The schematic diagram of the carriers’ motion under illumination (**a**) when *V_app_* < 0 (**b**) when 0 < *V_app_* < *V_bi_*.

Combined with the conductance G as well as photocurrent, the underlying process can be revealed as follows, which can be distinguished by five regions:

(i) At reversed bias more than 2 V, along with the increase of conductance, the capacitance increased slowly as well. Here, the reversed field can both dissociate excitons and drive carriers to electrodes. As a low hole mobility of Alq_3_, it would bring more charges’ accumulation in bulk, which may lead to the rise of C and also the space charge field *V_sp_*. As to G, its increase is due to the enhanced transport of electrons with increasing mobility of field-dependence [41]. Nevertheless, the presence of space charges will count against the conductivity leading to the slow increase of G. The photocurrent exhibited in Figure 2d also indicates the increasing reversed current in this region. Herein, although the current is dominated by the drift current *J_drift_*, we cannot neglect the influence of the diffusion current *J_diff_*. Since the holes stored near anode while electrons drifted to cathode, it would eventually give rise to gradient so as to form diffusion current *J_diff_* as shown in Figure 3a, which was against to *J_drift_*.

(ii) When operating under −2 V, the current reached 0. It can attribute to the equilibrium between *J_drift_* and *J_diff_* as mentioned above since *J_drift_* was weakened with the decreased field. Then decreasing the bias to 0 V, as can be seen, C increased slowly while G maintained in a constant low conduct state, 7 μS. As to G, under weakened field, *J_diff_* will overwhelm *J_drift_* to form forward current as shown in Figure 2d. The current was mainly composed of holes and thus resulted in a low conductance. In addition, the total current increased with the decreasing reverse voltage, which is nearly linear the inset in Figure 2d and obeys ohm’s law. Thus, G will remain constant. With respect to C, it mainly derives from the diffusion of holes and can be taken as the diffusion capacitance *C_diff_*. Since *C_diff_* is related to the concentration of carriers, the more diffusing holes can lead to the increase of C. However, the diffusion is too weak to dramatically enhance C.

(iii) When forward bias was applied, G increased gradually and finally reached its maximum at built-in potential, 2 V. However, C kept increasing until 1.6 V and then decreased. In this region, *V_bi_* will be partly offset by the forward bias, and the total potential (*V_tot_* = *V_bi_* − *V_app_* − *V_sp_*) will be reduced. With its decreasing, the drift of carriers will be weaker so as to accelerate diffusion as well as G. As to the electrons of higher mobility, with the decrease of field, the transport of them will be impacted remarkably so as to form more resident in bulk as Figure 3b shows, which will augment the gradient so as to enhance diffusion. In this case, however, the diffusion of electrons will dominate in *J_diff_* as the higher diffusion coefficient than holes. Hence, as the bias increased, the conductance G and photocurrent both increased. As to C, the diffusing electrons will recombine with some holes however C continued to increase. Thus, the dominate capacitance here is depletion capacitance *C_dep_* described by

(2) $$C_{dep}=A\frac{\varepsilon \varepsilon_{0}}{d}$$

A is the area of device, *ε*_0_ is permittivity of vacuum, *ε* is dielectric constant, and d is the width of depletion zone, which follows the relation of d under forward bias as:

(3) $$d\infty {\left(V_{bi}-V_{app}-V_{sp}\right)}^{1/2}$$

Thus, d would be reduced with increasing bias leading to the increase of *C_dep_*, which also corresponds to the recombination of holes. Here, *V_sp_* is taken into account as well. Compared with dark capacitance, it may mainly lead C to increase dramatically under illumination. As can be seen, the increase of C lasted for a while until the bias reached to 1.6 V. Commonly, the depletion capacitance would diminish when the depletion region collapsed, i.e., flat band was achieved [29]. Hence the decrease of C can account for the vanishing of field as *V_tot_* = *V_bi_* − *V_app_* − *V_sp_* = 0, and *V_sp_* here is 0.4 V. It is worth noting that these immobile space charges should be distinct from stored holes in diffusion. It is reported that the Alq3-based device has localized state of 0.25 eV after aging [52]. When operating under ambient atmosphere, our device can also suffer from aging. For the immobile space charge, it may trap in deep site for 0.25 eV or even more. For the mobile stored holes, it may trap in shallow cite less than 0.25 eV. Thereafter, however, G continued to increase to 2 V, which meant the immobile space charges against G were eliminated. In details, the field turned to forward and thus afforded to the drift of carriers. Holes will be driven to flow out while electrons will drift to anode to recombine with stored holes, resulting in the increase of G.

(iv) When the bias reached 2 V, the real flat band can be achieved for the absence of stored holes. With the bias increasing to 3 V, more photo-carriers would generate, and a low injection of electron would take place. However, the latter is too weak to dominate the current. As a result, the drift of photo-carriers possessed the current so as to be linear with applied bias, which obeyed Ohm’s law as shown in Figure 2c. Hence, G remained constant in this region. As to C, its slowly rather than suddenly decrease may be still owing to the collapse of depletion region. It should account for the spatial redistribution of *V_bi_* due to the interface [29]. Also, it was reported that during charge collection the inductive behavior would yield the negative contribution to the capacitance [42].

(v) When the bias was more than 3 V, along with more photo-carriers, the enhanced injection increased the conductance G and photocurrent, which also gave rise to the second capacitance peak. However, it was lower than the first peak. The reason is that the photo-carriers corresponding to the second peak was less than the stored charges corresponding to the first peak. In addition, the injection of carriers will neutralize parts photo-carriers. Therefore, the rise of the second capacitance peak is limited.

**Figure 4:**
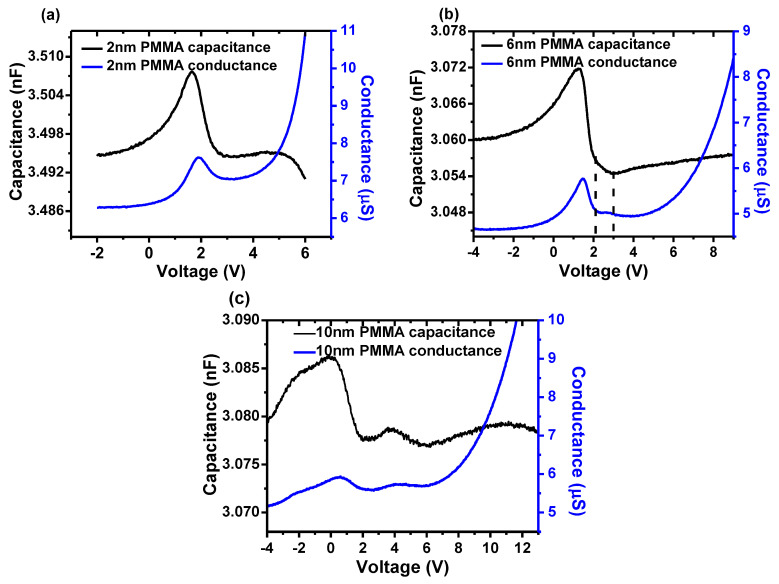
The C-V and G-V characteristics under illumination of devices with PMMA thickness of (**a**) 2 nm (**b**) 6 nm (**c**) 10 nm.

By analyzing the optical capacitance and conductance of the control device, it is concluded that the charges stored in bulk are attributed to the first capacitance peak. To achieve more stored charges, the insulator PMMA was inserted between PEDOT:PSS and Alq_3_. To our knowledge, 2 nm PMMA enables more injection of holes but less blocking of electrons. 10 nm PMMA shows the opposite way. 6 nm PMMA behaves moderately. Thus, the thickness of PMMA was chosen as 2 nm, 6 nm and 10 nm. The C/G-V characteristics of each device are shown in Figure 4. The total capacitance *C_tot_* can be equivalent to series capacitance between the capacitance of PMMA *C_PMMA_* the capacitance of Alq_3_ *C_Alq3_*, which can be described as follows,

(4) $$\frac{1}{C_{tot}}=\frac{1}{C_{PMMA}}+\frac{1}{C_{Alq3}}$$

Assuming both *C_PMMA_* and *C_Alq3_* are geometric capacitance, *C_Alq3_* is far more than *C_PMMA_* since the thickness of Alq_3_ is larger than that of PMMA. Thus, the capacitance of Alq_3_ dominates in the total capacitance. Given the depletion capacitance of Alq_3_, *C_tot_* can still be taken over by *C_Alq3_*. Thus, the influence of capacitance induced by PMMA can be disregarded.

As to the device with 2 nm PMMA, the peak to C and G located in 1.6 V and 2 V, respectively, which indicated no more stored charges. In addition, the optical capacitance exhibits a similar profile to that of the control device. However, the increase of conductance G took place under reverse bias. Between 2 V and 3 V, G alternatively decreased and the corresponding capacitance showed a deeply decrease compared with that of the control device. All these changes can originate from the influence of PMMA. As to ultrathin PMMA of 2 nm, it cannot effectively store charges so as not to change the first peak. However, it may exert negative influence on the carrier transport. When the device operated under reverse bias, the weakening hole drift due to PMMA may do favor to the diffusion so as to increase the conductance. When operating bias between 2 V and 3 V, the drift of electrons was confined and eventually decreased the conductance. The deeply decreased capacitance in this range may be due to the inductive behaviors of part confined electrons [42], which would count against the collection of carriers and thus contribute negatively to capacitance.

For the device with 6 nm PMMA, the peak of capacitance turned to lower bias 1.2 V and then the capacitance decreased greatly, even exceeded the minimum capacitance under reverse bias. It is found that the rate of decrease of capacitance was slower from 2 V to 3 V. With respect to the conductance, it began to increase under reverse bias and reach its peak at 1.6 V with a decrease thereafter. When running from 2 V to 3 V, it remained as a constant. Then it followed by gradual increase.

As the results show, with the thickness of PMMA increased, great changes occurred to the optical C/G-V characteristics of the device with 6 nm PMMA. However, the difference between the peak of C and G is 0.4 V, which is identical to that of 2 nm PMMA device, indicating the field of space charges *V_sp_* is not changed. However, the peak of C and G both shifted 0.4 V to lower bias, which accounts for the influence of 6 nm PMMA with more charges confined by it. As mentioned above, the maximum capacitance at 1.2 V means the flat band achieved. Then the maximum conductance was realized at 1.6 V indicating the elimination of space charges in bulk. However, there is still 0.4 V deviated to *V_bi_*. Furthermore, a great decrease of capacitance took place from 1.6 V to 2 V, which is reported to be related to the change of field induced by interfacial charges [44]. These charges originated from the holes captured by PMMA under reverse bias. When operating bias is more than 1.6 V, the drift electrons were confined by PMMA leading to the decrease of conductance. Meanwhile, these electrons will cut down interfacial charges so as to redistribute the spatial field, which influence the capacitance to decrease. When interfacial charges are all eliminated, the real flat band is achieved at 2 V. As to these charges, it was verified they are most trapped at shallow states in our previous work [29]. Then the capacitance decreased more slowly between 2 V and 3 V. In addition, the corresponding conductance turned to constant instead of decreasing. These changes may imply the formation of a new capacitance due to stored electrons. However, this capacitance is too weak to overwhelm the decrease of depletion capacitance. As a result, it just slowed down the decrease of capacitance.

To gain more evidence for the stored charge, the device with 10 nm PMMA was investigated as shown in Figure 4c. The capacitance exhibited three peaks. The first peak located at 0.1 V and the last one located at 10.8 V, which is corresponding to the depletion capacitance of stored charges and the diffusion capacitance of excess carriers, respectively. When running under reverse field, 10 nm PMMA in the device would store more holes at interface leading to more deviation of first peak. Additionally, it would prohibit charges injection so as to shift the last peak to a higher bias. As we expected, a new capacitance peak occurred between the first and last peak, which originated from the storage of electrons as mentioned above. With respect to the corresponding conductance, it can be found that G was in line with C when increasing as well as decreasing. Herein, the change of conductance should account for tunneling of stored charges. The tunneling current can be expressed as follows [43],

(5) $$J_{e}=\upsilon_{e}\sum_{e}\exp\left(-\frac{\Delta e}{F}\right)$$

Here, *υ_e_* is attempt-to-escape frequency for electrons, Σ*_e_* is the surface density of accumulated electrons, Δ*_e_* is the barrier height, *F* is the field in bulk. For tunneling current, it should be enhanced under a strong field. However, under lower bias, the surface density Σ*_e_* would mainly contribute to the tunneling. When interfacial charges were offset by confined electrons, extra field vanished. More storage of electrons will lead to its tunneling according to the Equation (3), finally resulting in the increase of capacitance as well as conductance. Nevertheless, more stored charges will bring a field against electron’s drift, limiting the increase of storage and the tunneling of charges. As a result, capacitance and conductance both decreased with it. Thus, the new capacitance peak is related to the stored electrons as speculated. Also, the capacitance induced by stored electrons can be approximately equivalent to diffusion capacitance as the similar process between diffusion and tunneling.

Thus, C-V combined with G-V can contribute to further investigate on underlying mechanism. This approach was used to analyze diode’s electrical characteristic based on impedance spectroscopy, i.e., *Y = G + jωC*. In our work, however, we were mainly dedicated to investigating the correlation between capacitance and conductance. With respect to our devices, the remarkable variation of capacitance and conductance commonly took place simultaneously, it thus can give specific evident of the charges’ motion. To our best knowledge, it is seldom used in IS analysis.

**Figure 5:**
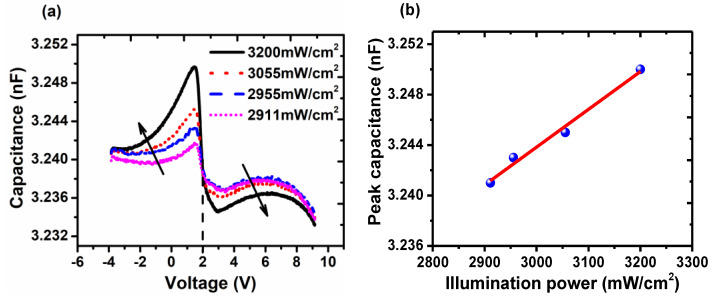
(**a**) The C-V characteristics for device with 6 nm PMMA under different illumination power (**b**) The relation between the value of first peak and illumination power.

To further investigate the mechanism, the capacitance under different illumination power was achieved in the device with 6 nm PMMA, which is shown in Figure 5a. As the power enhanced, the first capacitance peak increased correspondingly with a slight shift to lower bias. It should be derived from the more space charges induced by the intense illumination. As mentioned above, the stored charges induced by illumination can be taken as optical doping. More light power, i.e., more photon will produce more holes stored in localized states, which contributes to enhance the first peak and *V_sp_*. Also, when the device was illuminated in low power, the capacitance under reversed bias increased with the increasing bias, which is similar to that of the control device as shown in Figure 2c. The increasing capacitance originated from more stored charges, and it took place only when the drift carrier exceeded the diffusion carriers, i.e., the reversed field must be strong enough. Thus, the weak *V_sp_* would be prone to achieve strong reversed field resulting in its increase. With respect to the second peak, it increased with the weakened power and reached saturation at last. When *V_app_ > V_bi_*, photo-holes will be driven to cathode leading to the recombination with injected electrons. Thus, electrons would be reduced more under strong illumination, and the diffusion capacitance induced by electrons will be weakened. However, under weak illumination, the photo-holes were not enough to cut down electrons and thus capacitance did not increase any more. It is worth noting that all profiles crossed at 2 V and then decreased slowly. As mentioned above, the flat band can be achieved at 2 V, so all the values herein are identical. Thereafter, the storage of electrons could contribute slightly to the increase of capacitance. Also, the relation between the intensity of first peak and illumination power is shown in Figure 5b. As the result shows, the peak intensity is approximately in linear relation with the power, which is promising to get access to optical detections. Hence, it provides a new way to take advantage of stored charges.

## 4. Conclusions

In conclusion, we mainly investigated the stored charges in OLEDs on the optical C-V and G-V characteristics. Compared to the control device under dark, although C under illumination two peaks also appeared, the first peak of which was enhanced and deviated to *V_bi_*. Combined with G, it can be explained in the form of space charges stored in bulk, which would result in the local field *V_sp_* and impact on C. To achieve more storage of charges, devices with PMMA of different thickness were studied. As can be seen, as PMMA thickness increased, the first peak of optical capacitance would deviate more as well as decrease more during its collapse, which indicates that more holes are stored in interface. As to the device with 10 nm PMMA, an additional peak appeared between the first and last peak, which probably originate from the storage of electrons. By tuning the illumination power on the device with 6 nm PMMA, it is found that the more intense illumination power is, the more the capacitance peak increases, which reveals the contribution of stored charges to the capacitance under illumination. Meanwhile, it exhibits approximate linear relation between the intensity of peak and light power. Thus, the optical capacitance is promising to be used in optical detections and will pave a new way to take advantage of stored charges.
